# A method to identify positive tipping points to accelerate low-carbon transitions and actions to trigger them

**DOI:** 10.1007/s11625-025-01704-9

**Published:** 2025-08-07

**Authors:** Timothy M. Lenton, Thomas W. R. Powell, Steven R. Smith, Frank W. Geels, Floor Alkemade, Martina Ayoub, Pete Barbrook-Johnson, Scarlett Benson, Fenna Blomsma, Chris A. Boulton, Joshua E. Buxton, Sara M. Constantino, Sibel Eker, Kai Greenlees, Thomas Homer-Dixon, Kelly Levin, Michael B. Mascia, Femke J. M. M. Nijsse, Ilona M. Otto, Viktoria Spaiser, Simon Sharpe, Talia Smith

**Affiliations:** 1 Global Systems Institute, University of Exeter, Exeter, UK; 2 Manchester Institute of Innovation Research, The University of Manchester, Manchester, UK; 3 Eindhoven University of Technology, Eindhoven, Netherlands; 4 Edinburgh University Business School, Edinburgh, UK; 5 Institute for New Economic Thinking, University of Oxford, Oxford, UK; 6 SystemIQ, London, UK; 7 University of Hamburg, Hamburg, Germany; 8 Stanford University, Stanford, USA; 9 International Institute for Applied Systems Analysis (IIASA), Laxenburg, Austria; 10 Cascade Institute, Royal Roads University, Victoria, Canada; 11 Bezos Earth Fund, Washington DC, USA; 12 Sanford School of Public Policy, Duke University, Durham, USA; 13 Wegener Center for Climate and Global Change, University of Graz, Graz, Austria; 14 School of Politics and International Studies, University of Leeds, Leeds, UK; 15 S-Curve Economics, London, UK; 16 Circular Bioeconomy Alliance, London, UK

**Keywords:** Tipping point, Methodology, Transition, Decarbonisation, Acceleration, Action

## Abstract

Meeting the Paris Agreement to limit global warming to “well below 2 °C” requires a radical acceleration of action, as the global economy is decarbonising at least five times too slowly. Tipping points, where low-carbon transitions become self-propelling, could be key to achieving the necessary acceleration. We deem these normatively ‘positive’, because they can limit considerable, inequitable harms from global warming and help achieve sustainability. Some positive tipping points, such as the UK’s elimination of coal power, have already been reached at national and sectoral scales. The challenge now is to credibly identify further potential positive tipping points, and the actions that can bring them forward, whilst avoiding wishful thinking about their existence, or oversimplification of their nature, drivers, and impacts. Hence, we propose a methodology for identifying potential positive tipping points, assessing their proximity, identifying the factors that can influence them, and the actions that can trigger them. Building on relevant research, this ‘identifying positive tipping points’ (IPTiP) methodology aims to establish a common framework that we invite fellow researchers to help refine, and practitioners to apply. To that end, we offer suggestions for further work to improve it and make it more applicable.

## Introduction

Stopping global warming is essential to achieving sustainability. To meet the Paris Agreement to limit global warming to “well below 2 °C”, global anthropogenic net greenhouse gas emissions need to decline rapidly to zero, meaning the global economy needs to decarbonise at least five times faster than it is currently (Sharpe 2023; Lenton 2025). That in turn requires highly non-linear responses in the sectors and activities responsible for greenhouse gas emissions. Low-carbon transitions in a range of sectors need to accelerate rapidly to achieve a maximum rate of decarbonisation, before they inevitably slow as the last emissions are eliminated, following a declining ‘S-curve’. Tipping points where low-carbon transitions become self-propelling could be key to achieving the necessary acceleration and making it hard to reverse. We deem such tipping points normatively ‘positive’ because they can limit profound and inequitable harms from global warming.

The potential for tipping points in the adoption of innovative behaviours and technologies is well established from a range of historical case studies and associated theories, e.g. (Rogers 2003; Arthur 1989; Nakicenovic 1986). There is extensive empirical research on the diffusion of innovations (Rogers 1962, 2003) and several distinct mechanistic models identify tipping points defined in terms of a ‘critical mass’ of adopters (Zeppini et al. 2014). The ‘critical mass’ is the level of adoption in a population beyond which further adoption becomes self-propelling (or self-amplifying)—thanks to reinforcing feedback overwhelming balancing feedback (Mascia and Mills 2018). A tipping point can occur because adoption makes the new behaviour or technology more attractive to others, for example, through lowering its costs and/or improving its performance (Arthur 1989), or simply because we imitate or learn from others’ adoption (Rogers 2003).

Tipping points that markedly accelerated decarbonisation have been passed at national scales in electricity and auto-mobility sectors (Sharpe and Lenton 2021; Geels and Ayoub 2023). The UK power sector passed a tipping point for the elimination of coal power after 2012 (Sharpe and Lenton 2021; Lenton 2025), and a corresponding tipping point in the uptake of offshore wind power (Geels and Ayoub 2023; Lenton 2025), achieving a maximum decline of over 10% per year in CO_2_ emissions. The Norwegian car market passed a tipping point for the uptake of electric vehicles (EVs) around 2012 (Sharpe and Lenton 2021; Lenton 2025), triggering an accelerating decline in sectoral emissions. The uptake of solar photovoltaic (PV) power worldwide has recently passed a tipping point and is forecast to become the dominant source of power by 2050 (Nijsse et al. 2023). Criteria for identifying past social tipping points have also been proposed (Hodbod et al. 2024), recognising that social tipping points can be negative as well as positive (Spaiser et al. 2024).

A growing literature is starting to highlight the prospects for triggering further positive tipping points (Meldrum et al. 2023; Sharpe and Lenton 2021; Lenton 2020; Otto et al. 2020; Lenton et al. 2023a; FOLU/GSI 2021; Nijsse et al. 2024). A framework for ‘operationalising’ positive tipping points has been proposed that identifies a range of generic actors and actions that can bring them forward and trigger them (Lenton et al. 2022; Ong et al. 2023). Such practical guidance is of interest to many actors committed to accelerating emissions reduction. Notably, policymakers signed up to initiatives such as the Breakthrough Agenda, are seeking actionable policy advice to accelerate the transition. Leaders in the financial sector have formed groups aimed at shifting investment towards low carbon energy (e.g. the Glasgow Financial Alliance for Net Zero). Businesses have formed demand-side coalitions to procure net zero emission power, materials, and technologies (e.g. RE100, SteelZero, EV100).

Researchers are starting to propose specific actions to trigger specific positive tipping points (Sharpe and Lenton 2021; Meldrum et al. 2023; Lenton et al. 2023a; Nijsse et al. 2024; Muiruri et al. 2024; Chung et al. 2024). Analysis of past cases of positive tipping reveals complex sets of reinforcing feedbacks among different groups of actors (e.g. firms, users, policymakers, and wider publics)—and their actions—that can contribute to self-propelling change (Geels and Ayoub 2023). Yet, there is a lot more scope to provide targeted guidance. To move towards credible, actionable guidance it is essential to avoid wishful thinking about the existence of positive tipping points, or oversimplification of their nature, drivers, and impacts (Milkoreit 2023; Mealy et al. 2023)—because if the promised results do not occur this may demoralise key actors.

Not all past cases of socio-technological or social–ecological transitions involved tipping points. Numerous innovations have failed to diffuse or stagnated after initial acceleration (e.g. nuclear power). Other cases of accelerated adoption have subsequently been reversed (e.g. the reduction in traffic during the COVID-19 pandemic). Hence, we should not expect that all sectors and activities responsible for greenhouse gas emissions will have the potential to tip to a zero or low emission alternative. Learning rates and cost reduction speeds are lower for technologies with higher degrees of design complexity and customisation needs (e.g. nuclear power plants, carbon capture and storage, or building retrofits), weakening a key driver of positive tipping (Malhotra and Schmidt 2020). In some areas, positive tipping therefore requires us to direct innovation or deployment efforts towards modularity and scalability (e.g. mass customisation for building renovation), or to choose better technologies.

Mainstream economics assumes that the ‘marginal abatement cost’ increases with the proportion of emissions abated (i.e. it takes increasing effort to eliminate emissions the more are eliminated). We disagree with this as a general presumption, because cost is often a declining function of deployment (Way et al. 2022), producing situations where the more emissions are eliminated the cheaper it gets to eliminate the next unit (up to a point). Nevertheless, it is vital to distinguish cases where a tipping point is unlikely. For example, cement production is CO_2_ emissions intensive with no clear zero-emission substitute (yet), except carbon capture and storage (CCS), which has limited prospects or evidence (yet) for economies of scale or learning-by-doing dynamics.

Here, we propose a (prototype) methodology for identifying potential positive tipping points and actions that can trigger them, which is complementary to existing methods (Tàbara et al. 2022). The aim is to establish a common framework that a community of researchers can broadly agree upon and refine, and a range of practitioners (including policymakers and other actors) can apply. The paper is intended for both audiences. In the following, we first define a positive tipping point. Then we offer the methodology, framed in terms of a series of questions, and illustrated with case studies. We close by suggesting avenues for further work.

### Defining a positive tipping point

**Fig. 1:**
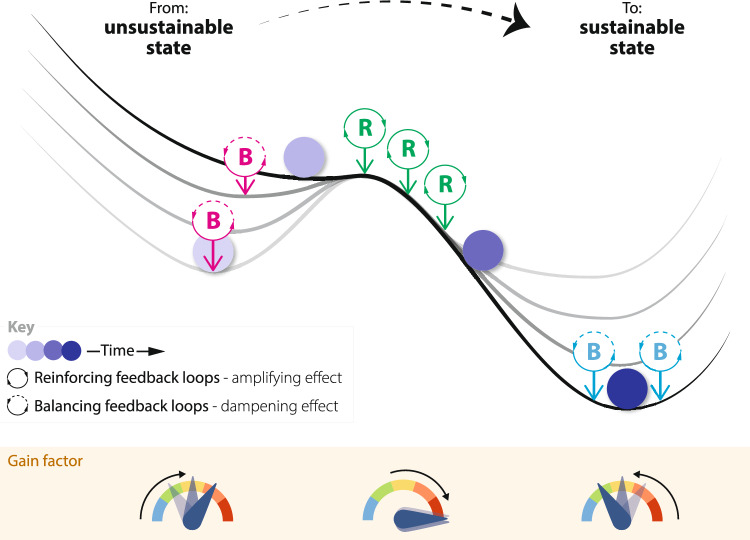
Schematic of a system going through a positive tipping point, showing the changing state of the system (ball) over time, influenced by a changing net effect of different balancing (B) and reinforcing (R) feedback loops in the system, summarised by the overall feedback ‘Gain factor’

A glossary is included as an appendix. We define a tipping point as the point beyond which self-propelling change occurs in a system, giving rise to a significant (qualitative) change in the system state (or attractor) (Fig. 1). Before the tipping point, there are balancing (negative) feedbacks that maintain the initial state of the system, but they are getting weaker. At the tipping point, self-propelling change is supported by reinforcing (positive) feedbacks within the system overwhelming these balancing feedbacks. Initially this produces accelerating change, but change will necessarily reach a maximum rate then typically slow down as a new system state is approached. In that new state, a new set of balancing feedback (‘lock-in’) mechanisms typically arise that stabilise it. Alternatively, self-propelling change can sometimes be stopped before there is a complete change in system state—as discussed further below.

For low-carbon transitions, which are shifts from fossil fuel-based systems to low-carbon systems (Geels et al. 2017), it is useful to distinguish two kinds of tipping points (Allen and Malekpour 2023): one—tipping toward—related to the accelerated diffusion of low-carbon innovations (which can be technologies, behaviours, or business models), and one—tipping away from—related to the abandonment of incumbent fossil fuel-based systems. Most of the literature has focused on the former, although the latter is essential to reduce greenhouse gas emissions. Both kinds of tipping points tend to be temporally related, in the sense that the accelerated diffusion of low-carbon innovations is often a significant contributor to the destabilisation and decline of incumbent systems (Turnheim and Geels 2012; Rosenbloom and Rinscheid 2020; Koretsky et al. 2022).

This paper is concerned with both kinds of tipping points, although—in line with the literature—we place greater emphasis on self-propelling uptake of zero or low greenhouse gas emission norms, behaviours, and products or technologies. In this case, a qualitative change is taken to mean that the adoption of the more sustainable norm, behaviour or technology ultimately extends to most of a population—although in some cases even a less extensive, initially self-propelling substitution may have sufficient impact on greenhouse gas emissions to warrant consideration. In the other case (tipping away from), a qualitative change means the abandonment of the existing unsustainable system by most of a population.

We consider both kinds of tipping points to be normatively ‘positive’ because of their potential to limit global warming and the harms and inequality it causes, by accelerating progress towards net zero greenhouse gas emissions. To reach net zero emissions also requires changes in land use and associated diets and farming practices, from being a net source to a net sink of greenhouse gases. Here there is also potential for positive tipping points towards innovations—e.g. the self-propelling diffusion of conservation initiatives (Mascia and Mills 2018; Mills et al. 2019; Clark et al. 2024)—and away from incumbent systems (e.g. the meat system).

There is a well-established (quasi-)linear relationship between cumulative (CO_2_-equivalent) emissions and global temperature change, and a huge literature linking global temperature change to different harms (IPCC 2022; Lenton et al. 2023b). Those harms are inequitable in being disproportionately caused by the richest (Chancel 2022; Rammelt et al. 2023) and falling disproportionately on the poorest (IPCC 2022; Lenton et al. 2023b), and that inequality is being exacerbated by global warming (Emmerling et al. 2024). Positive tipping points can therefore limit injustices and inequality being caused by global warming (Emmerling et al. 2024; Lenton 2025). We recognise that tipping away from incumbent systems and tipping towards new norms, behaviours or technologies can create justice concerns, and that weighing these up against the harms and inequalities being alleviated through reducing greenhouse gas emissions is critical to ensuring a just transition (Avelino et al. 2024; Pereira et al. 2024; McCauley et al. 2024; Gupta et al. 2023). Equally, rapid decarbonisation does not necessarily contribute to achieving other sustainable development goals or sustainability justice (McCauley et al. 2024). Therefore, the methodology offered should be nested within a broader, reflexive approach to achieving transformative change towards sustainability (Tàbara et al. 2019; Minna et al. 2024).

The initial state for an innovation—whether technological or behavioural (Törnberg 2018)—is typically one of occupying a niche, whereas for an incumbent system it is one of dominance. The progressive loss of balancing feedback that occurs before a tipping point is sometimes referred to as loss of resilience, in the sense of loss of ability of a system to recover from perturbations back to its original state. At and beyond the tipping point, where reinforcing feedback takes over, self-propelling change is initially self-accelerating, and often irreversible. However, a large and fast enough change in action (e.g. withdrawing key policy support) may be able to stop it. This can be seen as balancing feedbacks reasserting control (Ayoub and Geels 2024). In particular, tipping towards low-carbon innovation can give rise to balancing feedbacks that decelerate change, such as negative policy feedbacks (e.g. concerns over financial costs or distributional consequences leading to weaker policies), negative public debates (e.g. concerns about fairness or effects on particular social groups) (Ayoub and Geels 2024), or deliberate actions to oppose change by those with a vested interest in maintaining the status quo. However, if a new system state is attained, it can be stabilised by lock-in mechanisms including the availability of infrastructure, standardisation, norms and regulations for a new technology, and changes in societal norms for a new behaviour.

For positive tipping towards low-carbon innovations, there are multiple distinct feedback mechanisms that can independently give rise to self-propelling adoption and corresponding distinct models (Zeppini et al. 2014; Lenton et al. 2022) (Table 1). These different types of tipping mechanism are described in more detail elsewhere (Zeppini et al. 2014; Lenton et al. 2022)—and are complementary. Social contagion (Granovetter 1978), highlighted in diffusion of innovation theory (Rogers 1962, 2003), involves adopters imitating and learning from one another, without requiring any change in the thing being adopted (although adopters may tailor it to fit their needs). Increasing returns to adoption (Arthur 1989) involves adoption increasing the pay-off for subsequent adopters, because the thing being adopted may, e.g. get more affordable, attractive, and/or accessible through, e.g. learning-by-doing and economies of scale in its manufacture. In a coordination game, when a new suite of technologies (e.g. EVs and charging network) competes with an existing suite of technologies, the more adopters who coordinate on the new suite the better the return from further adoption (Kandori et al. 1993). Similarly, network effects occur when the more people who join a network (e.g. the telephone network), the more everyone gains from adoption. Information cascades can occur when information from others who have made adoption decisions overrides an individuals’ private judgement of the benefits of adoption (regardless of whose judgement is correct) (Bikhchandani et al. 1992). Percolation (Solomon et al. 2000) and co-evolution (Kauffman and Johnsen 1991) models are similar to the social contagion and coordination games, respectively. In real cases, a mix of types of tipping mechanisms may be at work, and additional types of feedback can play a role, e.g. policy feedback (Pierson 1993).

**Table 1:** Relevant models for tipping points towards low-carbon innovation, described in more detail elsewhere (Zeppini et al. 2014; Lenton et al. 2022)

Tipping model	Micro-foundations	Key positive feedback(s)	Form of tipping point	Control variables	Key reference	Sustainability examples
Social/behavioural contagion	Imitation, heterogeneous agents	Adoption makes it more likely for the next person to adopt	Critical mass	individual adoption threshold distribution, social network structure	Granovetter (1978)	Rooftop solar PV uptake, EV uptake, plant-based diet uptake, climate activism movement, conservation initiatives
Increasing returns	Utility maximisation, homogeneous agents	Technology becomes better and cheaper the more it is produced and adopted	Critical mass	Population size, difference in quality to incumbent technology, strength of increasing returns	Arthur (1989)	Solar PV, wind power, lithium-ion batteries, sharing economy (car sharing)
Coordination game (network effect)	Utility maximisation, homogeneous agents	Coordination on a new technology suite gives a superior payoff to an existing technology suite	Critical mass	population size, benefit, and cost of new technology, payoff from old technology	Kandori et al. (1993)	EVs and charging infrastructure (versus ICEVs and fuelling infrastructure)
Information cascades (herding)	Utility maximisation, heterogeneous agents	Adopter expectation based on previous adopter decisions	Critical mass	cost of adoption, probability of future gain, public information	Bikhchandani et al. (1992)	Organic farming
Percolation	Utility maximisation, heterogeneous agents	Word-of-mouth diffusion depends on neighbours’ willingness to adopt	Critical price	cost, number of starting seeds, network connectivity	Solomon et al. (2000)	Spread of The International Small-group Tree planting initiative (TIST)
Co-evolution	Utility maximisation, heterogeneous agents	Payoff depends on choices made by coupled others	Between fitness maxima	Number and fitness of components, degree of coupling, governance	Kauffman and Johnsen (1991)	Rooftop solar PV and grid storage, climate litigation, and financial risk to firms

Where the potential for a tipping point can be credibly established, sound advice on how it may be triggered must be grounded on clear understanding of its nature and drivers. Here, it is useful to distinguish the variable in which tipping occurs from the factors that can influence it and be influenced by it. The variable in which tipping occurs is often the population of actors adopting (or not) a new behaviour or technology (Rogers 1962, 2003; Zeppini et al. 2014) (Table 1); these actors can be consumers who purchase electric vehicles or adopt a plant-based diet, or firms that deploy wind or solar farms. The emerging literature sometimes considers qualities of what is being adopted—notably price (affordability), but also attractiveness and accessibility—as if they were the variable(s) in which tipping occurs. They are important factors to understand, insofar as they influence adoption decisions, but the risk here is assuming that if a criterion is met in one (or several) of them (e.g. price parity with incumbent technology), tipping (inevitably) occurs. It may do, but if adoption is held back by other factors, it can be delayed or prevented, or if adoption is pushed forward by other factors, it may happen beforehand. We instead argue for greater analytical clarity by distinguishing between adoption (in which positive tipping can occur), and factors that may influence adoption, and be influenced by adoption, and thus correlate with it. Clearer understanding and more nuanced guidance may emerge from this distinction.

For positive tipping away from incumbent fossil fuel-based systems, there are multiple amplifying feedback mechanisms that can accelerate breakdown (Turnheim and Geels 2012; Rosenbloom and Rinscheid 2020; Koretsky et al. 2022), involving increasing pressures on the existing system (e.g. from policymakers, civil society, technical alternatives, competitors), weakening performance (e.g. shrinking markets, financial losses, loss of legitimacy and licence to operate), and weakening confidence and commitment of incumbent actors (visible in less investment in the existing system and increased efforts at exploring alternatives). These mechanisms can further reinforce each other. Some can be seen as the same key reinforcing (positive) feedback as in tipping towards innovation but operating in reverse. Notably, technology can become less attractive as more people abandon it, economies of scale can work in reverse as production declines, and coordination games can break down—think of the escalating inconvenience of owning a petrol or diesel car as the fuelling and maintenance networks for it start to close. That said, the feedback mechanisms of tipping away from incumbent systems warrant better characterisation to draw up a comparable table.

**Fig. 2:**
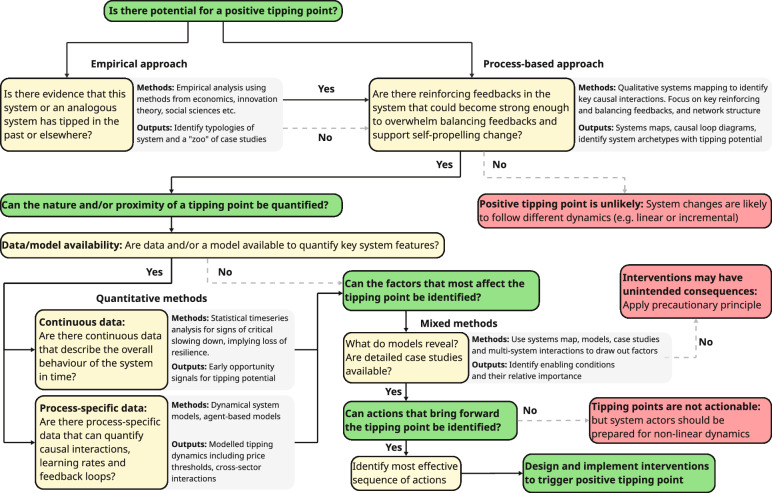
Summary flow diagram of overall methodology (noting that in practice it is more iterative than this linear depiction suggests)

Having defined a positive tipping point, we turn to how to identify the potential for one. Figure 2 summarises the overall methodology.

### Is there potential for a positive tipping point?

To identify the potential for a positive tipping point, a system of interest must be defined. This could be, for example, the global energy system, or a sector of the global economy responsible for significant greenhouse gas emissions (e.g. light road transport), or a sector in a particular country (e.g. the car market in China), or in a particular city (e.g. the mobility sector in Shanghai). Having identified the system there are, broadly speaking, two complementary approaches to identifying whether it has the potential for a positive tipping point.

The first approach is empirical—to consider existing experience and observations: is there evidence that either this system, or an analogous system, has tipped in the past, or elsewhere? If the system of interest has tipped in the past, then there is reason to think it may be capable of tipping in the future. For example, the contribution of coal burning to UK electricity production declined abruptly from 96% in 1956 to 77% in 1960, and again from 62% in 1991 to 33% in 1997, suggesting the potential for a further abrupt decline (as seen since 2012). However, past tipping is not a guarantee of the potential for future tipping, because changing boundary conditions can remove or create the possibility of tipping. The corollary of this is that just because a system has not tipped in the past does not mean it cannot tip in the future.

Inferring the possibility of tipping by analogy effectively takes the analogous system as a model for the system of interest—and we need to be careful (and self-critical) about our grounds for drawing the analogy, asking: do we think the two systems have similar qualities? For example, we may infer that because the UK power sector has tipped rapidly away from coal burning in the last decade, other national power sectors can exhibit such a tipping point. Some, such as Greece, Denmark, Portugal, Spain, and recently Chile, have done so (Jaeger 2023), but can we be confident that others can? China and India (in particular) are together responsible for two-thirds of the world’s coal power generation capacity today, have rapidly increasing electricity demand, and have much larger domestic coal mining industries than any of the countries that have tipped thus far. Clearly the differing development, size and structure of economies, and the differing nature of national electricity, political, and financial systems need to be considered. More generally, cultural dimensions (Hofstede 2011) may affect whether (and where) a positive tipping point can occur.

The second approach towards identifying a tipping point is more process based. It involves building a qualitative causal model of the system of interest to see if it has qualities associated with a tipping point. This can be done using systems mapping methods, especially causal loop diagrams (Barbrook-Johnson and Penn 2022; Eker et al. 2024; de Gooyert et al. 2024). Once the boundaries of the system of interest are established, the practice of systems mapping draws out key elements of the system, expressed as variables, and the causal interactions between them (Barbrook-Johnson and Penn 2022), while considering interactions with the wider context (Eker et al. 2024). Key elements may include qualities of incumbent and new behaviours or technologies (e.g. attractiveness, affordability, and accessibility), how they influence adoption, and how they are in turn influenced by adoption. A key step is to identify reinforcing (positive) and balancing (negative) feedback loops in the web of causal interactions. Of particular interest are reinforcing feedback loops that are known to be associated with relevant tipping dynamics, including learning-by-doing, economies of scale, technological reinforcement, and social contagion, and balancing feedback loops that are known to help maintain the status quo, e.g. ‘lock-in’ to existing behaviours or technologies with sunk costs (Levin et al. 2012), or subsidies and policy support for incumbents linked to lobbying and specific political agendas.

From existing system maps and/or causal loop diagrams, similarities can be sought between systems that are known to have had tipping points and our focal system—in particular, the potential for strong reinforcing feedbacks relative to balancing feedbacks. It may thus be possible to identify generic positive tipping system ‘archetypes’ (Stroh 2015).

The systems mapping approach can be usefully augmented in several ways (see next section), starting with looking for empirical evidence regarding the feedback loops identified. Additionally, considering system network structure (as distinct from feedback structure) can reveal structures that facilitate diffusion e.g. small-world networks (Newman and Watts 1999). Considering the strength and type of competition (or complementarity) between an innovation and the incumbent technology or behaviour can help identify cases of weaker competition that may lead to coexistence rather than a tipping point where an innovation displaces the incumbents (Geels and Schot 2007).

Combining these empirical and process-based approaches gives an initial assessment of level of confidence in the potential (or not) for a positive tipping point (Table 2). The empirical approach can suggest the possibility of tipping, but identification of a clear causal pathway is needed to ascribe high confidence in the potential for tipping. This includes identifying additional changes needed to stabilise the new system. The process-based approach may also reveal critical differences between existing cases and the system of interest that make tipping unlikely. Where no empirical evidence for past or analogous tipping can be found, the process-based approach may still suggest tipping potential, but with lower confidence, as this approach is strengthened when supported with data and insights from real cases. For example, empirical cases may reveal previously hidden or unexpected causal interactions, or factors that affect the strength of causal interactions. The combined approach cannot definitively resolve existence (or not) of a tipping point, but it provides a filter for what systems to consider in more depth. That invites a consideration of what data and modelling approaches are available to reveal more.

**Table 2:** Assessment of tipping potential and confidence thereof

Empirical approach	Process-based approach	Tipping potential	Confidence
Evidence exists for past or analogous tipping	Identifies mechanistic pathway for tipping	Yes	High
Evidence exists for past or analogous tipping	Suggests contextual differences or barriers to tipping in system of interest	Unlikely^a^	Medium
Evidence exists for past or analogous tipping	No clear functional causality path can be identified	Yes	Low
No evidence for past or analogous tipping	Identifies mechanistic pathway for tipping	Yes	Low/medium
No evidence for past or analogous tipping	Significant barriers to tipping or no mechanistic pathway identified	No	High

^a^Tipping may be possible if boundary conditions or some fundamental system interactions are changed

### Can the nature and/or proximity of a tipping point be quantified?

Having established a qualitative model of our system and assessed that it has potential for a positive tipping point, a next step is to consider what data and modelling approaches are available to try and get a quantitative sense about the existence, nature, and/or proximity of a potential tipping point. Data can be about the system’s overall behaviour (e.g. rising market share of EVs, declining market share of petrol/diesel cars, changing profitability of automakers), or more process-specific data about the strength of key interactions and feedback loops in the system and the factors they depend upon (e.g. how people’s adoption preferences change with factors such as price or adoption by neighbours). Process-specific data is a prerequisite for process-based modelling, where an important step is to evaluate whether existing models can be applied or extended to the focal case.

**Fig. 3:**
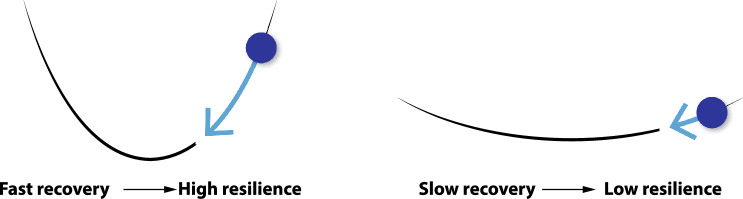
Schematic of critical slowing down before a tipping point (see Box 1)

If we only have data on the overall behaviour of our system—and must treat what goes on within our system as a ‘black box’—we can still learn something about whether our system may be prone to a tipping point or approaching one. Specifically, we can look for generic ‘early opportunity indicators’ that a tipping point is approaching, in the form of loss of resilience of the incumbent state of the system, defined as the ability to recover from perturbations (see Fig. 3, Box 1). Where there is data of a system being perturbed (e.g. the COVID-19 pandemic, price shocks, or new policy being implemented), we can directly observe the incumbent state’s response to learn about resilience. If it recovers rapidly from perturbation, then it is resilient, and the rate of recovery is a direct measure of resilience (faster is more resilient). If there are multiple perturbations, any change in resilience can begin to be assessed. Where sufficient time-series data is available we can derive continuous statistical indicators of (changing) resilience. Loss of resilience alone does not guarantee that there will be a tipping point to an alternative state or attractor. However, the nature of changing fluctuations in the system can give clues as to whether and what type of tipping point may be approaching (Kuehn 2011; Bury et al. 2021).

More system-specific early opportunity indicators can also be sought. The literature on destabilisation, decline, and phase-out (Turnheim and Geels 2012; Rosenbloom and Rinscheid 2020; Koretsky et al. 2022; Ohlendorf et al. 2022; Turnheim 2023; Blomsma et al. 2023) identifies specific causal mechanisms that may signal an approaching tipping point. These include increasing economic and socio-political pressures on the system, weakening competitive performance and eroding legitimacy, and increasing doubts by incumbent actors about the future viability of the existing system, leading to increased exploration of alternatives.

If we have process-specific data from our system, especially regarding the feedback loops identified by systems mapping, then we can use this to inform a more formal, quantitative model of the system. This may allow the existence (or not) of a tipping point to be more confidently established, the type of tipping point to be more precisely identified, and its proximity to be predicted. For example, a system dynamics approach can be used to create a stock and flow model that helps get a sense of inertia and lags that may affect system behaviour. Just quantifying the chain of causal interactions in a specific positive feedback loop can sometimes establish that amplification has the potential to get strong enough to produce a tipping point (by establishing that the ‘gain factor’ of the feedback loop is greater than or equal to 1). In cases of simple contagion, this is like assessing the ‘infectivity’ of a virus (Clark et al. 2024; Mascia and Mills 2018)—if it matches or exceeds 1, i.e. each carrier can infect at least one further person, a tipping point to an epidemic can occur.

For a complex contagion mechanism of tipping behaviour within a population, eliciting information (e.g. via surveys or social media data) on varying preferences across that population and what those preferences depend on (e.g. how much adoption is influenced by others adopting), can help quantify a potential tipping point (Peng and Bai 2024). Contagion mechanisms have played a key role in some past tipping points (Jagadish et al. 2024), but may be overemphasised in other cases (Smith et al. 2020). Other distinct generic types of tipping point model may be appropriate to adapt (Zeppini et al. 2014; Lenton et al. 2022), which differ in their assumptions about micro-foundations and mechanisms underlying tipping point behaviour (Table 1). Model choice therefore needs to be guided by empirical insights about the system and agents in question. Furthermore, it should be recognised that real-world tipping phenomena may involve a mix of the mechanisms that are captured in different simple models (and possibly other social mechanisms that are not captured in the simple models).

One generic model of competition dynamics between innovations and incumbents, which can provide a good fit to several empirical cases, are the Lotka–Volterra equations of population dynamics (Bhargava 1989; Morris and Pratt 2003). These equations allow for cases of abrupt tipping from one technology to another, or smooth tipping from one technology dominating to multiple technologies coexisting, depending on whether the competition between technologies is greater or less than the competition (between firms/brands) within a technology. For both types of tipping points (abrupt and smooth), early opportunity signals of loss of resilience of the incumbent technology are expected beforehand (van Nes and Scheffer 2007; Chisholm and Filotas 2009; Escobido and Hatano 2015). Mathematical analysis or training of a deep learning algorithm can offer the potential to distinguish between the cases (Kuehn 2011; Bury et al. 2021). The Lotka–Volterra equations allow for a mix of underlying feedback mechanisms to be at work without explicitly resolving their details. There are relatively few model parameters, offering the potential for calibration from data as a basis for (probabilistic) forecasting.

This is the mathematical basis of the Future Technology Transformations (FTT) model (Mercure 2012, 2018), which has been calibrated on empirical data for specific energy sectors—e.g. power, light-duty road transport—resolved at country levels. It has been used to (probabilistically) assess whether and where/when a tipping point of self-propelling uptake exists—for example, that a global tipping point for uptake of solar PV was recently passed (Nijsse et al. 2023). That said, the Lotka–Volterra approach assumes that the selection environment is homogeneous and the new therefore immediately competes with the old. Instead, radical innovations often emerge in sheltered niches where they do not immediately compete with the incumbent system (Raven et al. 2016). This may enable tipping points where innovations break out of niches and into mainstream markets to precede tipping points of destabilisation of an existing system.

More explicit and detailed models exist (or can be developed) for specific contexts, where there are sufficient data to justify it. Agent-based models, in particular, are increasingly used to study adoption dynamics of low-carbon innovations—for example, they have been calibrated on survey data to understand the controls on electric vehicle uptake in different jurisdictions (Kangur et al. 2017; Scorrano and Danielis 2022).

If we can develop a quantitative model of our system, then that can add to the qualitative systems mapping in helping identify what factors can affect a potential tipping point.

### What factors can most affect the tipping point?

Having identified the potential existence of a tipping point, and something about its nature (e.g. critical mass) and proximity, a key question is: which factors (variables) can most affect it? By factors here we mean things like affordability, attractiveness, and accessibility that can directly affect adoption (or not) of an innovation, or market size, profitability, and legitimacy that can affect abandonment (or not) of a fossil fuel system. These are all factors that may also be influenced by adoption (or abandonment), thus forming part of the feedback system. We treat deliberate actions (e.g. by policymakers) to affect such factors separately (next section), while recognising that they are also part of the system and subject to feedback (Geels and Ayoub 2023).

Qualitative systems mapping can be complemented by detailed case studies of the system in question, or a related one, to identify quantifiable and unquantifiable factors that can affect tipping. The latter include how issues and solutions are discussed, framed, and chosen in the political sphere. If our focal case can be likened to one or more of the generic models discussed above (Table 1), or if a more specific quantitative model of our system is available, that should encapsulate some of the controlling factors and provide information on their relative strength.

A set of properties that can enable (or inhibit) tipping of adoption has been drawn up from the set of generic models and some case studies (Lenton et al. 2022) (although additional insights from detailed case studies were no doubt missed). These factors include system properties of population size and social network structure, which can indirectly affect diffusion. Other factors that can directly affect adoption decisions include price (affordability), performance (quality), desirability (symbolism), accessibility (convenience), information (capability), complementarity (of technologies), and co-benefits (e.g. to health or energy security). These factors can all be endogenously shaped by actions and interactions in the system. Some are properties of the thing being adopted, some are properties of the adopters, and some are relational properties (between adopter and adopted, or between technologies).

Implicit here is that agents deciding whether to adopt (or not) are not perfectly rational actors, but they do exhibit bounded rationality (making decisions to satisfice, often against multiple criteria). Also, agents vary in their ‘appetite’ and reasons to adopt (i.e. populations are heterogeneous). For example, individuals’ adoption decisions may involve personal and cultural identity, their choice architecture, what others around them are doing, and factors such as health benefits, desire to help the environment, or other ethical concerns. Understanding of adopters and what influences them most can be obtained from, e.g. survey data (Kangur et al. 2017; Scorrano and Danielis 2022).

For any given case of adoption, the mix of properties affecting a tipping point, and their relative influence, may vary. Price (affordability) often gets most focus, but even if price were the only factor affecting adoption, a universal tipping point is unlikely. While global feedback mechanisms (such as declining price of lithium-ion batteries with increasing adoption) can tend to give a common tipping point, many localised factors can also affect the tipping point. For example, a tipping point to adoption of EVs was correlated with total cost of ownership parity in Norway (Figenbaum 2020), but in China was more associated with purchase price parity (Lenton 2025). For other technologies such as plant-based milk substitutes, price appears less important, as early adopters (with 15% market share in the USA) have been willing to pay twice the price of conventional milk. Identifying the balance of factors affecting tipping in any given case is a key part of the research agenda.

**Fig. 4:**
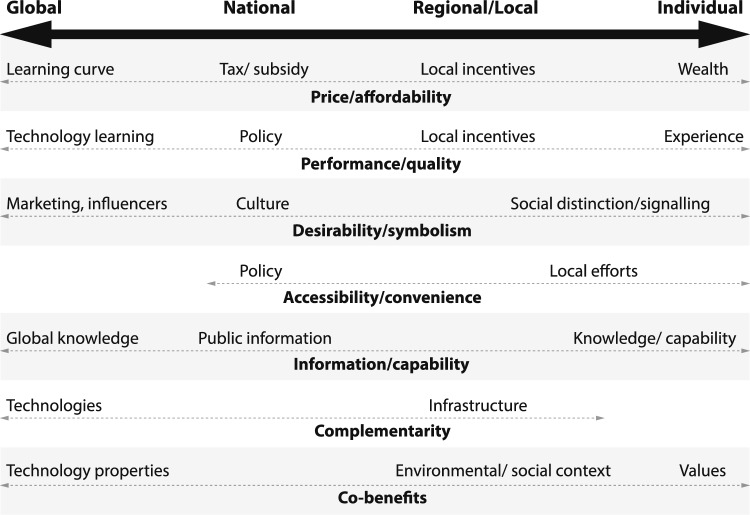
Schematic of how factors that can directly affect an adoption tipping point span spatial scales

A generic sense of how endogenous factors that can influence tipping vary across scales is summarised in Fig. 4. Price may vary from regional to (inter)national scales depending on, e.g. production and distribution costs, and taxes and subsidies or incentives. Learning curves in production costs extend to global scales and in distribution and installation costs down to local scales. Performance may vary with geography (e.g. wind turbines and solar panels depend on weather and seasonality). Learning-by-doing in product development can improve performance up to the global scale, and learning-by-using can improve performance down to local scales. Desirability depends partly on the adopter, e.g. wealthy individuals can value the symbolism of signalling their environmental values with purchases, but that symbolism also often depends on rarity, which declines with adoption. Accessibility (convenience) can depend on relevant infrastructure from national to local scales (e.g. EV charging stations) and on the adopters (e.g. whether they have a driveway on which to install a home EV charger). The complementarity of technologies is partly a property of technologies themselves, but realising their mutual reinforcement is sensitive to efforts at national to local scales (e.g. to establish a common standard for swappable EV batteries for 2- or 3-wheelers). Adoption decisions can be influenced—in either direction—by information, which can sometimes spread contagiously. Such information cascades can be initiated or stopped by public information coming from trusted bodies, across international to local scales. The capability to access information also varies among individuals. Perceived co-benefits (or detriments) of adoption are partly a property of behaviours or technologies, but also depend on individual worldviews and their social and environmental context.

Multi-system interactions between sectors or geographies (Rosenbloom 2020) can also influence a tipping point in a particular sector and geography. For example, stimulating innovation in a major market can bring down price and improve quality of a product in all markets. Equally, rapid adoption in one sector (e.g. Li-ion batteries in road transport) can bring forward tipping in another sector (e.g. batteries being used to balance renewable electricity supply and demand) (Nijsse et al. 2024). Also, some actors, institutions, and technologies (e.g. digitisation and AI) can have cross-cutting influence across multiple sectors. Systems mapping can start to identify key interactions and feedback loops between sectors (e.g. Fig. 5 in Meldrum et al. 2023). To quantify the strength of cross-sector interactions and feedback loops requires data. Detailed models can help quantify specific interactions, e.g. showing that second-hand EV batteries can meet demand for short-term grid electricity storage as early as 2030 (Xu et al. 2023). Resulting insights can then be incorporated in country-level macro-economic models like FTT (Nijsse et al. 2024). Quantifying the interactions between the power, light-duty road transport, heavy-duty road transport, and heating sectors in FTT shows that their tipping points reinforce one another (Nijsse et al. 2024).

If the factors that most affect tipping cannot be reliably identified, then a precautionary approach to intervention should be adopted—meaning that any deliberate action should proceed with caution and seek to maintain reversibility if unwanted consequences arise.

### What actions can most effectively bring forward the tipping point?

If the factors that influence a positive tipping point in a system can be reliably identified, then a crucial step is to identify what actions, by which actors, can affect these factors to most effectively bring forward the tipping point. Building on systems mapping and quantitative modelling, this can draw on multiple methods, including the leverage points framework (Leventon et al. 2021).

Policy actions are a key focus, because the overall goal is instigating accelerated change for the common (public) good. Equally, civil society actors and communication campaigns can build the social and political legitimacy for change, shifting the ‘Overton window’ of what policies are considered socially acceptable. Private sector actors can also invest in bringing forward tipping points that they expect to benefit from, or simply as a hedging strategy, while others may act to oppose tipping points that they perceive they will lose from. Coordination of actions by different actors intent on triggering tipping can be particularly effective (Lenton 2025).

Both empirical and process-based approaches can help identify interventions with the most potential to bring forward tipping points. Empirical analysis of past and recent social–technological tipping points already reveals some general lessons—notably, the most effective policy action tends to vary with proximity to a tipping point (Grubb et al. 2024). When combined with established theory, several generic reinforcing feedbacks between key actor groups can be identified (Geels and Ayoub 2023). For a target case of potential positive tipping, systems mapping can use this generic set of reinforcing feedbacks between actor groups (Geels and Ayoub 2023) as a partial guide, to help capture the interactions between actors and actions that can create (or oppose) change in a system. Social activists can be key to triggering early policy action, aided by the media (Lenton 2025; Nisbett et al. 2024). To have the potential for reaching a tipping point, in the innovation phase of a new technology, policy support for experimentation, R&D, skills development (for deployment), and creating niche markets of deployment (e.g. through public procurement) are usually important. Incumbent firms and private capital can also drive R&D investment—notably for alternative proteins (Mylan et al. 2023)—stimulated by consumers creating demand that drives further investment in a reinforcing feedback loop (Geels and Ayoub 2023). Feedback between policy and firms often reinforces such domestic growth of innovations (Geels and Ayoub 2023; Pierson 1993; Jordan and Matt 2014; Millar et al. 2021; Meckling 2019).

To break out of a niche and reach a tipping point, policies such as deployment subsidies (to ensure profitability), purchase incentives (to improve affordability), and infrastructure support can be critical. Regulations and mandates that force the private sector to reallocate investment from the old to the new technologies can drive innovation, economies of scale, and progress along learning curves, improving the attractiveness and/or affordability of the new technologies, e.g. for solar PV (Green 2019; Nemet 2019). As regulation of new technologies or behaviours requires legitimacy, this is typically only politically feasible when either the sense of urgency is high (e.g. COVID-19), the societal norm has already shifted (smoking) or the technology is widely available and affordable (zero emission zones). International cooperation can strengthen pertinent feedbacks (IEA et al. 2022), and these feedbacks can in turn increase the options for international cooperation (Meckling 2019), although unintended balancing feedbacks like the triggering of trade wars are harder to predict (e.g. EU-China over solar PV) (Meckling 2019). Monitoring and accountability mechanisms are important to tracking and ensuring progress (Mascia and Mills 2018).

Where a quantitative model captures the relevant processes, it can be used to begin to predict the impact of different policy actions. For example, the FTT model allows the effects of country-level policy actions—e.g. taxes, subsidies, regulations, mandates/phase-outs—to be evaluated individually and in combination. For example, FTT predicts that taxes and subsidies are insufficient to trigger a tipping point to EVs in India, but the addition of regulations and an EV mandate can tip the market (Mercure et al. 2024). Furthermore, mandates in one major market, e.g. increasing the ZEV share of sales towards 100% by 2035, can markedly bring forward a tipping point across markets—for example, a ZEV mandate in the EU and UK brings forward cost parity for EVs in India by a year (Mercure et al. 2024). Clean technology mandates can also bring forward tipping points in other coupled sectors (Nijsse et al. 2024).

Calibrated agent-based models that better capture social contagion and network effects in a particular population, can allow additional policies (e.g. investment in EV fast-charging infrastructure) and policy combinations to be evaluated (Kangur et al. 2017). However, quantitative models are only just starting to capture the political economy of change and resistance to it (Moore et al. 2022). Hence forecasts of what specific actions could achieve need to be nested within broader systems understanding of the political economy. Notably, actions by other actors that may seek to prevent or delay a tipping point must be considered, and policy designed accordingly. Here, a focus on policy feedbacks can help inform policy actions that create path dependency and lock-in to a new alternative while breaking out of lock-in to the incumbent way of doing things (Levin et al. 2012; Meckling and Karplus 2023).

## Application and future directions

**Fig. 5:**
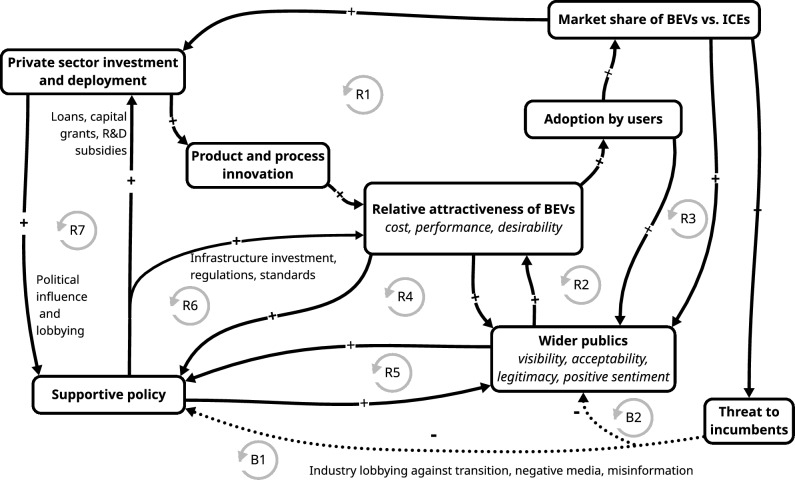
Systems map of key reinforcing (R) and balancing (B) feedback loops in the UK light-duty road transport system (see Box 2)

To illustrate the application of the methodology, we provide a case study (Fig. 5, Box 2). There are several avenues of further work that could refine the methodology, and aid its application by practitioners, which should be responsive to cultural context (Schneider et al. 2022). Starting with the research agenda and following the order of the methodology:

A key research target is to see if ‘archetypes’ of positive tipping can be identified. Notably, what does the pattern of positive feedback loops that destabilise the old system or reinforce the new system, and negative feedback loops that keep the status quo or maintain the new look like? Research is also needed to identify and classify specific causal mechanisms for tipping points of destabilisation and breakdown of incumbent fossil fuel-based systems (Turnheim and Geels 2012), which might usefully draw on, e.g. past abolition movements (Azar 2007).

Data-based methods of quantitatively assessing positive tipping points can be advanced. Causal inference methods applied to multiple data streams (where available) could help identify key interactions and feedback loops (Sugihara et al. 2012). Methods of resilience sensing from data to look for ‘early opportunity signals’ of the potential for a positive tipping point (Box 1) need further testing and development to better understand their applicability and utility. Initial tests have been restricted to the car market (Boulton et al. 2025; Mercure et al. 2024), but there is scope to extend to other sectors, e.g. solar PV uptake. There are minimum data requirements to reliably estimate resilience indicators, hence a systematic scan of available data across different sectors would help indicate where resilience sensing is possible in principle. Testing the significance of apparent trends in resilience against appropriate null models is also important.

There is considerable scope to further develop quantitative models of potential positive tipping points at different scales. This tends to be a specialist endeavour, but open sourcing model code with training/tutorials and guidance on appropriate use cases can help broaden the community of users. FTT is being extended to further sectors of the energy system and couplings between sectors are being added (Nijsse et al. 2024). Agent-based models (ABMs) can be applied to a wider set of cases where calibration data is available, including generative ABMs using large language models (LLMs) to simulate human behaviour (Lu et al. 2024). ABMs could be useful for understanding the combined effect on global markets of disparate national policies in sectors where competitive international trade is currently a barrier to the transition, including steel, chemicals, shipping, aviation, and agricultural commodities linked to deforestation.

The assessment of factors that can affect an adoption tipping point, in a particular cultural and geographic context (Hofstede 2011), can draw on decades of research on diffusion of innovations (Rogers 1962, 2003) and collective action (Hardin 1982). It is an area ripe for new research and increased cross-disciplinary collaboration with social scientists. For example, micro-economists and others already gather relevant data on individual preferences, which can be used to e.g. calibrate agent-based models. Experimental economists can design and run experimental tests of the influence of different factors on social tipping (Andreoni et al. 2021). Psychologists can experimentally study the effects of choice architecture on feedback and potential tipping dynamics (Brescia 2019). These fields mostly focus on short-term tangible factors. Therefore, social science approaches are also needed (like discourse theory, social movement theory, social constructivism) that analyse how more intangible factors like cultural meanings and interpretations of innovations (e.g. EVs, wind turbines, alternative proteins) and existing technologies (e.g. diesel cars, coal, meat) are shaped through longer-term public debates and framing struggles. This is increasingly being tracked through analysis of social media (Nisbett and Spaiser 2023). Such research can help provide a refined list of factors that can affect tipping in a specific sectoral, geographic and cultural context, and a sense of their relative importance.

Turning to practitioners, to help them empirically identify opportunities for positive tipping points it would be useful to build up an online resource of relevant, illustrative examples. The *ecotippingpoints.org* website already provides a valuable library of positive tipping points in (localised) social–ecological systems, which could be usefully extended to case studies of positive tipping in social–technological systems. This could be communally assembled by diverse groups as a ‘wiki’ resource with some editorial quality control. To aid practitioners in identifying if there is the potential for a positive tipping point in specific cases, a primer on tipping mechanisms would be a useful reference guide. This would describe key types of tipping mechanisms to look for, and their different underlying reinforcing and balancing feedback mechanisms (with diagrams). Participatory systems mapping workshops can help put such resources into practice, helping practitioners map out and understand their system of interest (Barbrook-Johnson and Penn 2022). Such an early stage of co-production can also be used to make value laden choices in the methodology explicit, helping strengthen its credibility and uptake (Partelow et al. 2025). Research efforts to publicly track and visualise the relevant factors affecting tipping could provide a novel source of information to actors in a system. Together with ‘early opportunity signals’ from resilience sensing, this could encourage actions to trigger a positive tipping point (Andreoni et al. 2021) (although we recognise that, conversely, discouraging information could deter social action). The identification and implementation of actions to bring forward positive tipping points would benefit from further co-production with practitioners, notably policymakers, but also firms, investors and social activists. Once key actions have been identified for specific positive tipping points, tracking the status of those actions across relevant sectors, geographies, and cultures could again provide a valuable source of information feedback to actors in the system, helping them prioritise deployment of finite effort and resources.

Overall, the methodology would benefit from more explicitly considering equity and justice issues throughout, both in terms of its procedure and the content (Tàbara et al. 2024). It could also be usefully augmented by addressing the question: What actions can delay or prevent the tipping point? While the positive tipping point concept is seductive, we need to put a different ‘thinking cap’ on (de Bono 1985) to recognise what can oppose tipping and strategise accordingly. This can address specific questions, such as: is it possible to circumvent or undermine the proposed solution? If so, what can counter that? In a complex adaptive system, interventions can trigger novel responses and game theory applies. One way to unpack how the dynamics could play out is with horizon scanning exercises with key stakeholders.

## Conclusion

We have proposed a methodology for identifying potential positive tipping points, assessing their proximity, identifying the factors that can influence them, and the actions that may bring forward positive tipping. Our aim has been to establish a common framework that a community of researchers can further refine, and a range of practitioners can start to apply—as action to find and trigger positive tipping points is now imperative.

Box 1: Early opportunity indicators Generic methods for detecting when a system is approaching a tipping point come from dynamical systems theory and have primarily been applied to ecological and climate systems (Scheffer et al. 2009; Dakos et al. 2024). They are based on the theory of critical slowing down (CSD) (Wissel 1984); the phenomenon that as a system approaches a tipping point, where the incumbent state loses stability, it will start to respond more sluggishly to external perturbations as the balancing feedbacks that maintain the status quo start to weaken. In a system with identified external perturbations, either the return time or return rate to the quasi-equilibrium state can be measured each time (Boulton et al. 2025). An increase in return time or slowing of return rate would suggest that the current state of the system is losing resilience and may be approaching a tipping point. Figure 3 shows a schematic of the concept of CSD for ‘potential wells’ that describe the strength of the balancing (negative) feedbacks in the system. In a system that is more resilient and further from tipping, the balancing feedbacks of the system cause a fast recovery (denoted by the steeper sides of the potential well). As resilience is lost, the potential well shallows, representing the weakening of balancing feedbacks in the system, hence the recovery from perturbations is slower.

For systems where external perturbations are not always clear or easily identified, changes over time in statistical properties of the system can indicate changes in resilience. Notably, theory predicts that lag-1 autocorrelation (AR (1)) and variance should increase in proportion on approaching a tipping point, due to CSD (Ditlevsen and Johnsen 2010). They can be measured on a sliding window across the time series of the system. For this method to work requires that (i) data exists that is representative of the dynamics of the system, (ii) it is of high enough temporal resolution to resolve the timescale of the system, (iii) it is of long enough duration to resolve any changes in that timescale, and (iv) it is not driven by highly autocorrelated noise. These criteria can make the approach challenging for social systems, where data is often limited.

Nevertheless, there are cases meeting the data requirements and the methods have been applied to social systems including social media (Pananos et al. 2017), financial crises (Wen et al. 2018), and disease outbreak (Proverbio et al. 2022). Recently, we have applied them to car market share (Mercure et al. 2024) and advert view (Boulton et al. 2025) data. We find that fluctuations in the market share of internal combustion engine vehicles (ICEVs) show CSD prior to abrupt declines in market share in Chinese and European markets (starting in 2020), while the US market shows neither CSD nor an abrupt decline (Mercure et al. 2024). We also find that the rising EV share of advert views of secondhand cars in the UK (and conversely the declining share of ICEV advert views) is exhibiting CSD, notably spikes of interest in EVs are becoming larger and more persistent (Boulton et al. 2025).

Box 2: Worked example: EVs in the UK. Here, the variable of interest is the share of EV sales in the UK car market.

**Is there potential for a positive tipping point?**

*Is there evidence that this system or an analogous system has tipped in the past or elsewhere?* Yes: there are historical cases of rapid conversion from horse and cart to ICEVs, and recent cases of rapid ICEV to EV market shifts, notably in Norway (and some other European countries).

*Are there reinforcing feedbacks in this system that could become strong enough to overwhelm balancing feedbacks and support self-propelling change?* Yes: the UK light-duty road transport system exhibits several reinforcing feedbacks (Geels and Ayoub 2023) including (Fig. 5): (i) increasing returns to adoption (learning-by-doing, economies of scale, technological reinforcement, e.g. an expanding charging network) (R1); (ii) percolation/diffusion of pro-EV information and social norms (R2 and R3); (iii) reinforcing policy feedbacks (e.g. zero emission vehicle mandate increasing supply of EVs) (R4–7). There is also political momentum (e.g. for public health and climate action). However, balancing feedbacks are resisting a tipping point, including lobbying by incumbents with interests in delaying or halting the transition (B1), and misinformation campaigns critical of EVs supported by right-wing newspapers, politicians, and social media influencers (B2).

Assessment: yes, a positive tipping point is possible. Potential: high.

**Can the nature and/or proximity of a tipping point be quantified?**

*Are there continuous data that describe the overall behaviour of the system in time?* Yes: early opportunity signals show critical slowing down and loss of resilience in the UK ICEV regime, as seen in both ICEV market share (Mercure et al. 2024) and advert view data (Boulton et al. 2025).

*Are there process-specific data that can quantify causal interactions, learning rates, and feedback loops?* Yes: the data-calibrated future technology transformation (FTT) model shows the importance of the learning rate of declining battery (and thus EV) price with increasing production (Mercure et al. 2024). Agent-based models of populations tipping to EVs also exist (Kangur et al. 2017).

Assessment: yes, data shows evidence that the UK car market is approaching a tipping point and modelling can assess its proximity.

**Can the factors that most affect the tipping point be identified?**

*What do models reveal? Are detailed case studies available?* Several factors can be identified: Affordability: a new EV was 36% more expensive than an ICEV equivalent in 2023, but total cost of ownership for a mid-sized car became comparable around 2024, and FTT modelling predicts that price parity will be reached around 2026 for mid-sized cars and 2028 for luxury vehicles. Attractiveness: average range of BEVs grew 75% between 2015 and 2023 with improvements in battery technology and speed of charging increased from 50 kW in early 2010s to up to 350 kW in 2024 (IEA 2024). Accessibility: better infrastructure promotes EV adoption (Buhmann and Criado 2023), but 47% of UK consumers believe there are too few charging points (Autotrader Consumer Research, May 2023). Information promotes EV adoption (Buhmann and Criado 2023). Co-benefits of cleaner air encourage uptake elsewhere (He and Zheng 2024).

**Can actions that bring forward the tipping point be identified?**

Zero emission vehicle mandates and efficiency regulations are likely to be the most cost-effective policies for driving the transition to EVs worldwide, with subsidies and taxes having additional roles to play (Mercure et al. 2024). The UK’s mandate towards 100% of new car sales being zero emission by 2035 is therefore important, albeit vigorously opposed by right-wing newspapers. Stronger policy support for public and home charging, grid expansion, and incentives e.g. free parking, could enhance EV uptake. Changes to building regulations to mandate chargers (as proposed by the EU) would aid adoption especially for those in rented accommodation. Coordinated international policy action especially zero emission vehicle mandates in larger markets (China, EU and USA), can significantly bring forward the tipping point in the UK (and other smaller markets) (Mercure et al. 2024).

## Data Availability

This study did not generate any new data.

## Glossary

Attractor: A state or set of states towards which a system tends to evolve for a wide range of initial conditions.
Contagion: The spread of a phenomenon or behaviour through a population. In simple contagion, spread only requires contact with a single agent; in complex contagion, spreading requires multiple contacts with multiple agents.
Critical mass: A type of tipping point in a social system where one extra person adopting a new technology, idea, or behaviour causes everyone else to adopt.
Critical slowing down: A phenomenon in which the speed of a system’s recovery from perturbations slows down before a tipping point. This feature of certain systems can be exploited to create early indicators of tipping.
Diffusion of innovation: A process by which new technologies, ideas, products, or services spread through social systems over time, typically in a non-linear, S-shaped trajectory.
Early opportunity indicator/signal: A statistical indication that a system is approaching a positive tipping point, usually due to critical slowing down.
Enabling condition: A system condition (for example, price parity) that can allow a positive tipping point to be triggered.
Feedback mechanism/loop: A closed causal loop within a system whereby an initial change feeds back to dampen/balance (mathematically negative feedback) or to amplify/reinforce (mathematically positive feedback) the change.
Intervention: A deliberate input to a system designed to influence the enabling conditions, feedbacks, or triggers for a positive tipping point.
Low-carbon transition: Transition from fossil fuel-based system to a low-carbon system.
Niche: A protected space where technological innovations can gestate without facing mainstream market competition. Also applies to social innovations.
Non-linearity: A situation where the output from a system is not directly proportional to the input.
Path dependence: A situation where past events constrain future events.
Percolation: A phenomenon caused by adding or activating nodes or links to a network, whereby the network abruptly becomes globally connected, allowing change to rapidly spread, whereas previously it was locally contained.
Positive tipping point: A tipping point that is predominantly beneficial to human health and well-being (and to ecosystem health), sometimes referred to as ‘social tipping point’ and ‘positive social tipping point’.
Qualitative change: The appearance or disappearance of important features and change in the balance of feedbacks. When quantifiable, it refers to a (much) larger change than a system’s standard deviation. When non-quantifiable, it refers to a change considered fundamental or important according to a normative judgement.
Resilience: The capacity of a system to resist or absorb change while continuing to function in its present state. When quantifiable, it is defined as the capacity of a system to return to its original state after perturbation.
Self-propelling: Change in a system that continues even when forcing is removed, until a different stable state is reached.
Social system: Human system.
Social–ecological system: A coupled system of social (human) and ecological components.
Socio-technical system: A system that includes people, technologies, markets, firms, infrastructures, policies, and supply chains.
Stable state: A state to which a system will return for some range of initial conditions or perturbations. Stability is maintained by balancing feedbacks that resist change.
System: A group of interacting or interrelated things that act according to a shared set of rules to form a recognisable unity.
Tipping dynamics: Changes in a system over time that result from crossing a tipping point.
Tipping point: A point (threshold) beyond which self-propelling change occurs in a system, giving rise to a significant (qualitative) change in the system state. The resulting change can be abrupt and/or irreversible.
Transition: A process of managed, often sector-specific socio-technical change.
Trigger: A change that causes a system to cross a tipping point.
